# Chromatin Compaction Protects Genomic DNA from Radiation Damage

**DOI:** 10.1371/journal.pone.0075622

**Published:** 2013-10-09

**Authors:** Hideaki Takata, Tomo Hanafusa, Toshiaki Mori, Mari Shimura, Yutaka Iida, Kenichi Ishikawa, Kenichi Yoshikawa, Yuko Yoshikawa, Kazuhiro Maeshima

**Affiliations:** 1 Structural Biology Center, National Institute of Genetics, Mishima, Shizuoka, Japan; 2 Frontier Research Base for Global Young Researchers, Graduate School of Engineering Osaka University, Suita, Osaka, Japan; 3 Radiation Research Center, Osaka Prefecture University, Sakai, Osaka, Japan; 4 Department of Intractable Diseases, Research Institute, National Center for Global Health and Medicine, Shinjuku, Tokyo, Japan; 5 Inorganic Analysis Laboratories, Toray Research Center, Inc., Otsu, Shiga, Japan; 6 Advanced Radiation Biology Research Program, Research Center for Charged Particle Therapy, National Institute of Radiological Sciences, Inage, Chiba, Japan; 7 Department of Physics, Graduate School of Science, Kyoto University, Kyoto, Japan; 8 Research Organization of Science and Engineering, Ritsumeikan University, Kusatsu, Shiga, Japan; 9 Department of Genetics, School of Life Science, Graduate University for Advanced Studies (Sokendai), Mishima, Shizuoka, Japan; National Cancer Institute, United States of America

## Abstract

Genomic DNA is organized three-dimensionally in the nucleus, and is thought to form compact chromatin domains. Although chromatin compaction is known to be essential for mitosis, whether it confers other advantages, particularly in interphase cells, remains unknown. Here, we report that chromatin compaction protects genomic DNA from radiation damage. Using a newly developed solid-phase system, we found that the frequency of double-strand breaks (DSBs) in compact chromatin after ionizing irradiation was 5–50-fold lower than in decondensed chromatin. Since radical scavengers inhibited DSB induction in decondensed chromatin, condensed chromatin had a lower level of reactive radical generation after ionizing irradiation. We also found that chromatin compaction protects DNA from attack by chemical agents. Our findings suggest that genomic DNA compaction plays an important role in maintaining genomic integrity.

## Introduction

Genomic DNA is wrapped around histones to form a nucleosome structure [1] [2] [3]. Although the higher-order chromatin structure in eukaryotic cells is not fully understood, several reports, including our recent cryo-microscopy and synchrotron X-ray scattering analyses, have demonstrated that chromatin consists of irregularly folded nucleosome fibers (10-nm fibers) in cells [4] [5] [6] [7] [8] [9] [10]; for review see, [11] [12]. Based on these studies, we suggested that interphase chromatin forms numerous compact chromatin domains, which resemble “chromatin liquid drops”, in the interphase cells [5] [9]. This view is in line with the predictions of the chromosome territory-interchromatin compartment (CT-IC) model [13] [14]. In the CT-IC model, each CT is built from a series of interconnected, megabase-sized chromatin domains, which were originally identified using pulse labeling as DNA replication foci [15] [16] [17] [18] that were shown to persist stably during subsequent cell generations [19] [20] [21]. Recent high-throughput 3C studies such as Hi-C and 5C have also proposed the physical packaging of genomic DNA which has been termed “topologically associating domains” [22], “topological domains” [23], or “physical domains” [24].

Although chromatin compaction is essential for mitosis to maintain the integrity of genomic information, whether compact chromatin domains confer other advantages, particularly in interphase cells, has not been elucidated. In previous *in vitro* studies, DNA compaction was shown to play a key role in protection against double-strand breaks (DSBs) generated by γ-rays [25] [26] [27] [28]. Therefore, we explored the significance of higher-order chromatin structures in the DSB generation process. Left unrepaired, DSBs caused by radiation can lead to chromosome fragmentation, chromosome loss, and the rearrangement of genetic information, events that are frequently associated with tumor formation and progression [29] [30]; also, see [31].

Much is already known regarding the mechanism(s) of DSB repair [29] [30]; however, little is known about how chromatin structure influences DSB induction processes, especially the quantitative and mechanistic aspects [32] [33] [34], although the involvement of reactive hydroxyl radicals in the induction of DSBs has been suggested [35] [36] [34] [37] [38]. Whether sensitivity to DSB induction differs for “open” chromatin configurations and inactive “condensed” regions has not yet been resolved [39] [40], because of the following reasons: *In vivo*, difficulty in the manipulation of chromatin structure, the lack of an efficient damage detection system, and regional differences in DNA repair efficiency have precluded drawing a decisive conclusion. *In vitro*, since long genomic DNA can be damaged during experimental manipulations, no efficient *in vitro* system has been developed for the manipulation of long chromatin and the quantitative detection of generated DSBs.

In the present study, we developed a novel system for chromatin manipulation and sensitive DNA damage detection, and succeeded in quantifying the DNA damage caused by ionizing irradiation. Importantly, the frequency of radiation-induced DSBs in fully decondensed chromatin was 5–50-fold higher than that in the condensed chromatin, indicating the existence of a DNA damage protection mechanism that is mediated by higher-order chromatin.

## Results

### Development of a Novel System for Chromatin Manipulation and DNA Damage Detection

**Figure 1:**
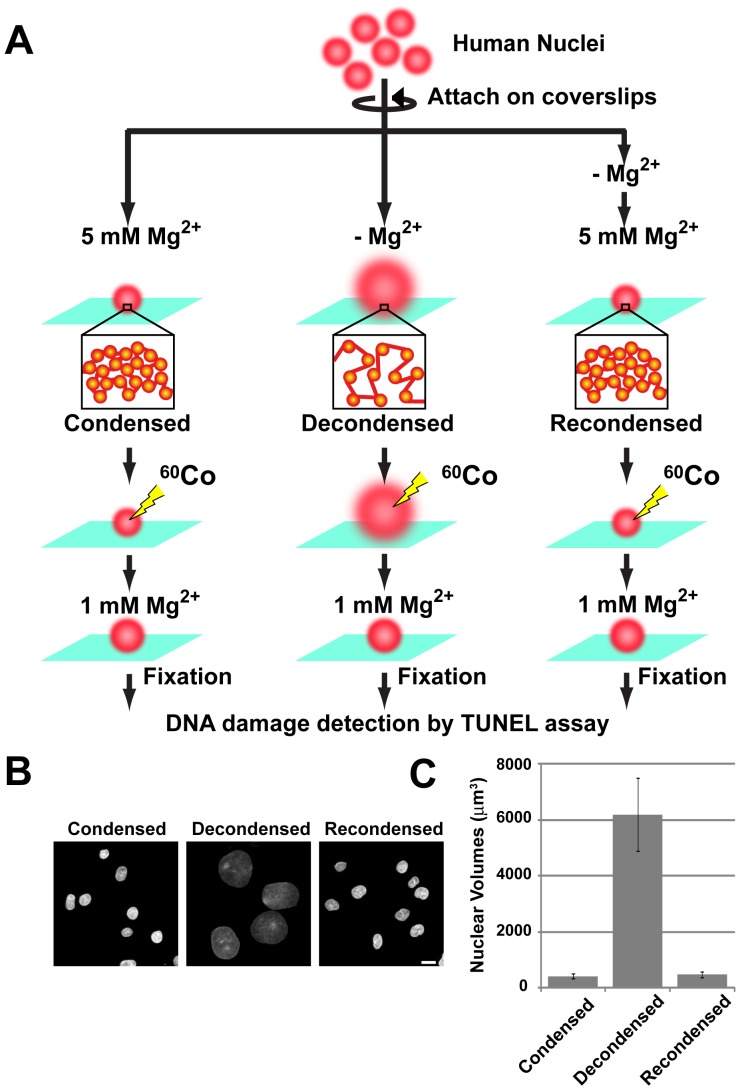
Schematic representation of the “solid-phase” system for chromatin manipulation and DNA damage detection. (**A**) The nuclei and their chromatin on the glass surface became condensed in the presence of 5 mM Mg^2+^ (left, see also Panel B) and decondensed in the absence of Mg^2+^ (center, see also Panel B). With increasing Mg^2+^ concentrations, the decondensed nuclei and chromatin recondensed (right, see also Panel B). Since chromatin compaction by cations is a type of phase-transition process [41], the two conditions (with 5 mM Mg^2+^ and without Mg^2+^) are sufficient to reconstruct the chromatin condensation and decondensation states. The three types of nuclei were irradiated with cobalt-60 γ-rays at varying dosages. The irradiated nuclei were treated with a buffer that contained 1 mM Mg^2+^, and the DSBs in the chromatin were detected using a TUNEL assay [43] (bottom). (**B**) Microscopic images of the condensed, decondensed, and recondensed nuclei with DNA staining (DAPI). Bar, 10 µm. (**C**) Volumes of the condensed (417.7±91.0, N = 50), decondensed (6196.6±1310.3, N = 50), and recondensed nuclei (Recondensed; 478.6±98.7, N = 50) (see the *Methods*). The standard deviation is shown as an error bar.

To examine whether the higher-order chromatin structure is directly involved in the induction of DNA damage (e.g., following exposure to γ-rays), we developed a novel system for chromatin manipulation and DNA damage detection (Figure 1A). To analyze DNA on the genome scale without causing physical damage, we used permeabilized nuclei that were attached to glass surfaces. The nuclei were isolated from HeLa cells and attached to poly-l-lysine-coated coverslips by gentle centrifugation (Figure 1A). Since chromatin is negatively charged, the compaction states of the nuclei and chromatin were controlled by altering the Mg^2+^ concentration in the environment; at 5 mM Mg^2+^, chromatin becomes highly condensed, whereas it decondenses in the absence of Mg^2+^ [41] [42] (Figure 1A). Based on the nuclear volume and known size of the genomic DNA, we estimated the DNA or chromatin concentration in the environment. This “solid-phase system” allowed us to perform very gentle and quantitative handling of the genome-sized DNA (Figure 1A). For the direct detection of DNA damage in the chromatin on the glass surface, we fluorescently labeled the DSB sites using terminal deoxynucleotidyl transferase (TdT) (i.e., a TUNEL assay [43]) (Figure S1). Thus, we detected DSBs in the chromatin without inducing additional breakages, which distinguishes our method from traditional gel electrophoresis-based assays (Figure 1A).

### Chromatin Compaction Protects Against DNA Damage by γ-ray Irradiation

As shown in Figure 1B, the nuclei and associated chromatin on the glass surface were condensed in the presence of 5 mM Mg^2+^ (∼400 µm^3^). In the absence of Mg^2+^, the nuclei and chromatin swelled or decondensed by approximately 15-fold (∼6000 µm^3^) (Figure 1B and C). To check whether decondensation could induce DNA damage, we recondensed the nuclei and chromatin (Figure 1B) to a size comparable to that of the condensed nuclei (∼450 µm^3^) by increasing the Mg^2+^ concentration (Figure 1C).

The three types (condensed, decondensed, and recondensed) of nuclei were irradiated with various doses of cobalt-60 γ-rays (Figure 1A). To promote the formation of a uniform compact state, which ensured equal chromatin accessibility and handling among the three types, the irradiated nuclei were treated with 1 mM Mg^2+^ then fixed with formaldehyde (Figure 1A). As a result, the nuclei became similar in size. DSBs in the chromatin were then detected using a TUNEL assay (Figure S1).

**Figure 2:**
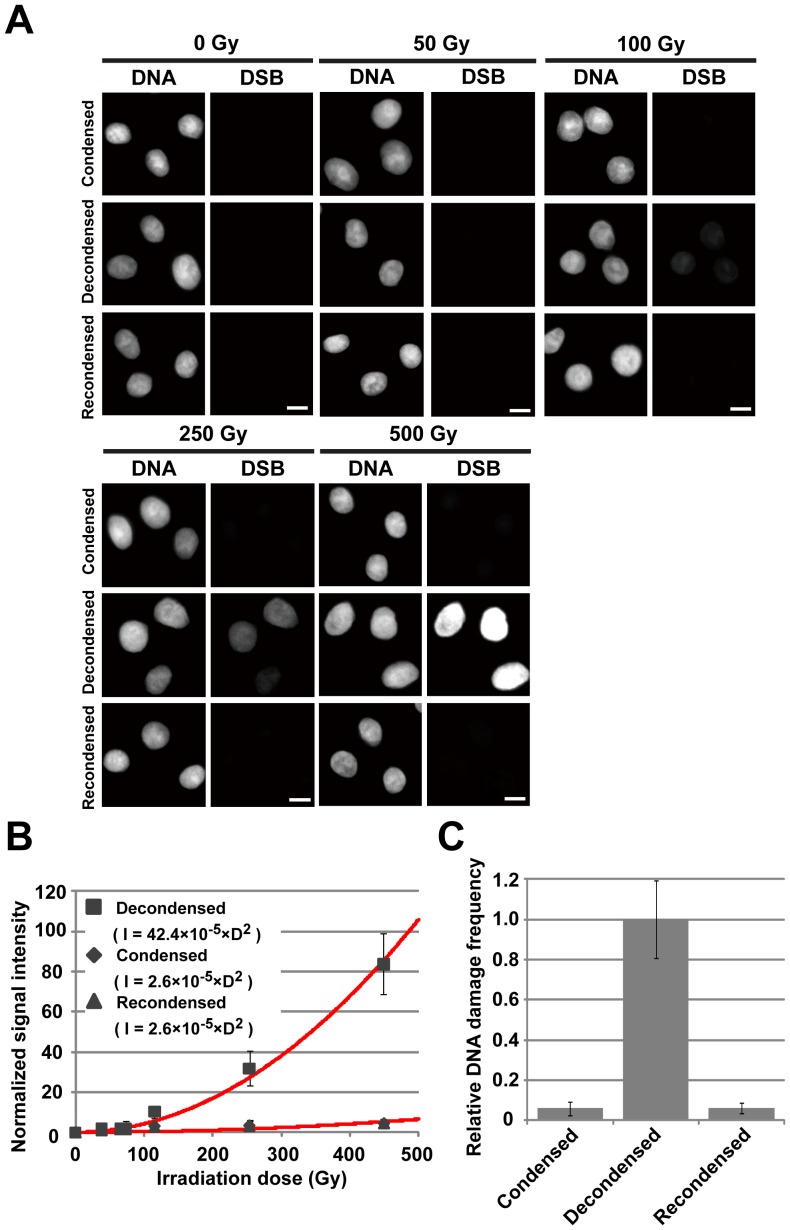
Chromatin compaction suppresses DNA damage caused by γ-ray irradiation. (**A**) DSB signal detection based on a TUNEL assay. For each radiation dose, DNA staining (left) and DSB signals (right) are shown. In the decondensed nuclei (2^nd^ row), a prominent increase in DSB signal was observed in a dose-dependent manner. However, the condensed (1^st^ row) and recondensed (3^rd^ row) nuclei showed only faint signals. Note that their sizes became similar because the three types of irradiated nuclei were treated with a buffer that contained 1 mM Mg^2+^, so as to drive them into a uniformly compacted state and ensure equal chromatin accessibility and handling among the three types. Bar, 10 µm. (**B**) Quantification of the detected DSB signals. The plotted normalized signal intensities and irradiation doses fit well to the quadratic curve I = kD^2^, where I, D, and k are the DNA breakage by irradiation, irradiation dose, and constant, respectively. The formula for each condition is shown in the graph. For each point, N >150. (**C**) The relative DNA damage frequency upon exposure to 500 Gy of irradiation for each condition is shown as a bar graph (Condensed, 0.06±0.03, N>100; Decondensed, 1.0±0.19, N>100; Recondensed, 0.06±0.03, N>100). The error bar shows the standard deviation.

As shown in Figure 2A, while the DSB signal intensity in the decondensed chromatin increased in a radiation dosage-dependent manner, the condensed and recondensed chromatin showed only faint signals. The suppressive effect observed for the recondensed chromatin excluded the possibility that the DNA damage was caused by the decondensation process (Figure 2A). In addition, we obtained similar results using a comet assay, which is a widely used damage detection method based on agarose gel electrophoresis [44] (Figure S2), although stronger DSB signals were observed even in the condensed chromatin, probably due to the non-specific breakages during manipulation steps (e.g., nuclear embedding in hot agarose). Taken together, these results indicate that condensed chromatin is much more resistant to γ-ray irradiation than decondensed chromatin.

### The Damage Signal Intensities Fit Well to a Quadratic Curve

We next quantified the DSB signals (Figure 2B). The signal intensities fit well to a quadratic curve in this irradiation dose range: I = kD^2^ (I, DNA breakage; k, a constant; and D, the irradiation dose). By comparing the k values, we found that the condensed chromatin had 16-fold greater damage suppression effects than decondensed chromatin (Figure 2C). The quadratic curve suggests that DSB induction is caused by two independent single-strand DNA breaks (SSBs). Consistently, under the assumption that DSBs occurred when two random SSBs were generated within ten bases in the double strands (Figure S3A), a simulation also showed that the number of DSBs created increased quadratically with the number of SSBs generated (Figure S3B).

### Protection Against Damage in the Low-dose Range of Irradiation

**Figure 3:**
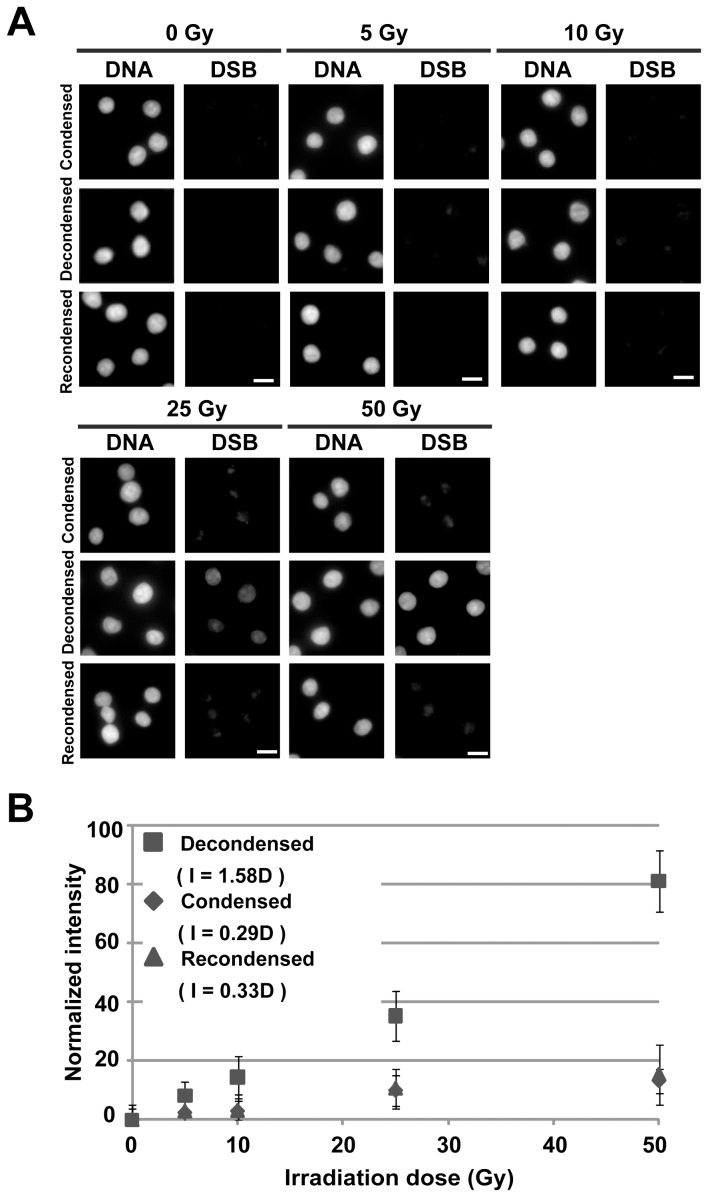
DSB damage protection effects in the low-dose range. (**A**) For the sensitive detection of DSB signals, an EMCCD camera was used. For each radiation dose, DNA staining (left) and DSB signals (right) are shown. Note that the DSB signals in the low-dose range are readily detectable in the decondensed chromatin (2^nd^ row). Bar, 10 µm. (**B**) Quantification of the detected DSB signals in the low-dose range. Normalized signal intensities and irradiation doses are plotted. The error bar shows the standard deviation. Consistent with our high-dose irradiation results, the condensed chromatin had considerable damage-suppressive effects. Note that the DSB signals seem to increase linearly at a low dose of irradiation.

We next focused on a lower dose range of irradiation (<100 Gy). To detect DSBs, we used an electron-multiplying charge-coupled device (EMCCD) camera, which can detect single photons. Using the EMCCD, DSB signals within the low dose range were readily detectable (Figure 3A). We found that the condensed chromatin had ∼5-fold more damage suppression effects, even at 5 Gy of irradiation (Figure 3B). Moreover, in contrast with high-dose irradiation, the damage frequency increased linearly, suggesting that the DSBs were formed in a single step, which is consistent with the notion that DSBs are induced linearly with irradiation dose [31] [37] (see also the *Discussion*).

### The Protective Effect on Condensed Chromatin is Related to the Chromatin Concentration, but not the Level of Chromatin-associated Proteins

Previous studies have suggested that chromatin-associated proteins (non-histone proteins) are involved in the induction of DNA damage [32] [33] [34]: the more proteins carried by the chromatin, the more protected the chromatin is against ionizing irradiation. However, we found that the total level and composition of associated proteins were similar between decondensed and recondensed chromatin, indicating that the protective effect on condensed chromatin is not due to the level of associated proteins (Figure S4). Moreover, no significant irradiation-induced degradation of proteins was observed (Figure S5). These results demonstrate that chromatin compaction is important for the protection of genomic DNA against ionizing irradiation.

**Figure 4:**
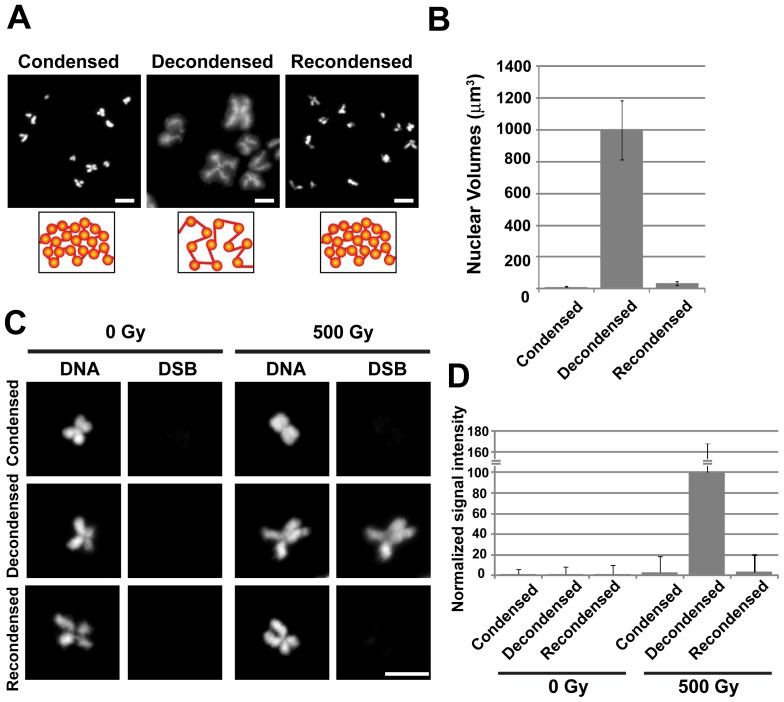
Chromatin compaction in mitotic chromosomes suppresses the DNA damage caused by γ-ray irradiation. (**A**) Microscopic images of condensed (left), decondensed (center), and recondensed (right) mitotic chromosomes with DNA staining (DAPI). Bar, 10 µm. (**B**) Volumes of the condensed, decondensed, and recondensed chromosomes (see the *Methods*). The standard deviation is shown as an error bar. (**C**) DNA damage detection using a TUNEL assay. For 0 Gy of irradiation (left two columns) and 500 Gy of irradiation (right two columns), DNA staining (left image) and DSB signals (right image) are shown. Bar, 10 µm. In the decondensed chromosomes (2^nd^ row), a strong increase in DSB signal intensity was observed. However, the condensed (1^st^ row) and recondensed (3^rd^ row) chromatin showed only faint signals. (**D**) Quantification of the detected DSB signals. The DNA damage frequency for each condition is shown as a bar graph. The error bar represents the standard deviation.

To confirm these observations, we performed similar experiments using isolated human mitotic chromosomes [45]. In the absence of Mg^2+^, the mitotic chromosomes swelled approximately 50-fold, as compared with those in a compact state (Figure 4A and B). The results obtained using chromosomes were more striking than those obtained using nuclei, in that the compact chromatin had approximately 50-fold greater resistance to γ-ray irradiation than the decondensed chromatin (Figure 4C and D). As the chromosomes contained fewer non-histone proteins than the nuclei (Figure S6), they were under fewer physical constraints and were more decondensed in the absence of Mg^2+^. These results provide further evidence that the protective effect is due to the chromatin concentration (volume) rather than the number of chromatin-associated proteins.

### Chromatin Compaction through Molecular Crowding also has a DNA Damage-suppressive Effect

**Figure 5:**
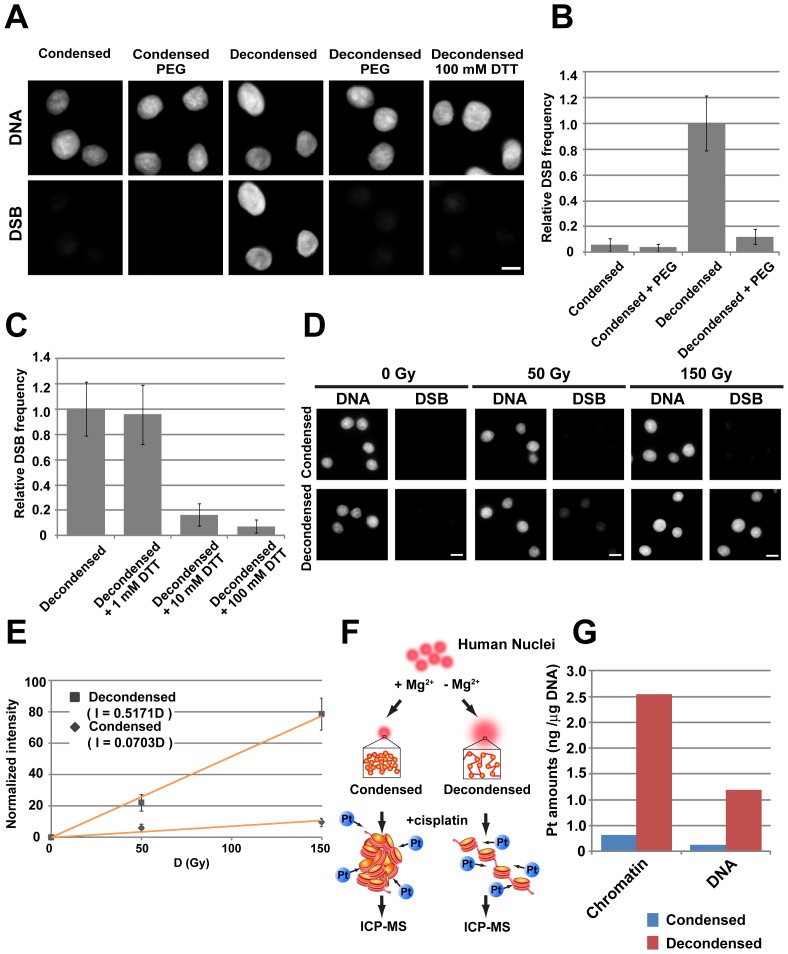
Effects of molecular crowding and radical scavengers on DNA damage suppression and protection from attack by heavy ions or chemicals. (**A**) DSB signals in the chromatin samples under various conditions. For each condition, DNA staining (1^st^ row) and DSB signals (2^nd^ row) are shown. The addition of PEG or DTT to the decondensed nuclei had strong DNA damage-suppressive effects. Bar, 10 µm. (**B and C**) Quantification of the DSB signals after the addition of PEG or DTT. The error bar represents the standard deviation. For each point, N = ∼300. (**D**) DSB damage protection against heavy ion irradiation. For each radiation dose, DNA staining (left) and DSB signals (right) are shown. (**E**) Quantification of the detected DSB signals. Normalized signal intensities and irradiation doses are plotted. The error bar shows the standard deviation. Note that the DSB signals increased linearly under this condition. Bar, 10 µm. (**F**) Treatment of condensed and decondensed chromatin with cisplatin. Schematic representation of the experiment. (**G**) The amount of cisplatin bound to the chromatin (nuclei) and extracted DNA was quantitatively measured by ICP-MS. Note that the DNA in the decondensed chromatin had 10-fold more cisplatin adducts than the condensed chromatin.

We next used polyethylene glycol (PEG) instead of Mg^2+^ to induce chromatin condensation, since a high concentration of macromolecules (100–200 mg/ml) in cells might condense chromatin through a molecular crowding effect [46] [47]. The addition of 12.5% PEG increased the compaction of decondensed chromatin, with a 5-fold nuclear volume reduction (Figure S7). We found a notable inhibitory effect (Figure 5A and B). We also considered that PEG might be contributing to the damage inhibition effect not only as a molecular crowding agent, but also as a scavenger of radicals (see below).

### Radical Scavengers Inhibit DNA Damage Caused by Ionizing Irradiation

We demonstrated that the chromatin concentration plays a critical role in the suppression of DNA damage by ionizing irradiation. Since reactive radicals arising from the radiolysis of water molecules might be a major contributor to DNA damage [34] [35] [36] [37] [38], a lower concentration of chromatin, which entails more water molecules and reactive radicals, could increase the risk of attack by radicals. Thus, we examined the effects of the radical scavenger dithiothreitol (DTT) on DNA damage. When the decondensed chromatin was irradiated in the presence of DTT, DNA damage was suppressed in a dose-dependent manner (Figure 5A and C). Since the sizes of the decondensed nuclei in the presence of 100 mM and 0 mM DTT were similar (Figure S7), the inhibitory effect observed in the presence of DTT was due to radical scavenging rather than chromatin compaction.

### Chromatin Compaction Protects Genomic DNA Against Heavy Ion Irradiation and Chemicals

We next considered whether the protective effect of chromatin compaction is limited to γ-ray irradiation. To address this question, we examined the protective effect against heavy ion (carbon ion beam) irradiation. Carbon ion radiotherapy is an emerging modality in cancer therapy, and its clinical applications are increasing worldwide [48]. We found a 7-fold higher damage suppressive effect in compact chromatin compared with decondensed chromatin (Figure 5D and E). This demonstrates that chromatin compaction protects DNA not only from γ-rays, but also heavy ions.

We also tested the protective effect of chromatin compaction against chemical attack. Since we used ethylenediaminetetraacetic acid (EDTA) for chromatin decondensation, DNA cleavage reagents, which often contain metal ions, were not applicable to our study. Instead, we used cisplatin, which is widely used as an anti-tumor drug [49]. Cisplatin forms covalent adducts with genomic DNA, thereby interfering with DNA replication and/or transcription, and eventually leading to apoptotic cell death. We treated the compact and decondensed chromatin with cisplatin, extracted the DNA, and measured the number of cisplatin adducts by inductively coupled plasma mass spectrometry (ICP-MS) (Figure 5F). We found that the DNA in the decondensed chromatin had 10-times more cisplatin adducts than the condensed chromatin (Figure 5G), suggesting that chromatin compaction protects genomic DNA against chemical-induced damage.

## Discussion

In the present study, we found that condensed chromatin had 16-times (nuclei) and 50-times (chromosomes) more resistance to γ-rays than decondensed chromatin, demonstrating that damage induction depends on the chromatin concentration (or volume) (Figures 2, 4, and 5). Importantly, this dependency suggests that the contributions of Mg^2+^ and other components in the system to the suppression of damage are negligible. As suggested previously [28] [37] [50] [51], the damage-suppressive effect of compaction is likely because the higher-concentration (compacted) chromatin has fewer water molecules per chromatin, thereby generating fewer reactive radicals. Compared with previous studies, which suggested that relaxed nuclear chromatin has 3.1–4.5-fold more DSBs than compacted chromatin [32] [33] [34], our results are particularly striking because the solid-phase system we used generates fewer background signals (non-specific DNA breakages) and allows for greater chromatin decondensation than agarose-embedded cells. Moreover, we were able to evaluate the suppressive effect in a quantitative fashion, as described below.

Using low-dose γ-ray irradiation, the DSB frequency increased linearly (Figure 3B), in contrast to high-dose irradiation. This suggests that the creation of DSBs by γ-ray irradiation involves a single step and two independent steps (“DSB efficiency” = a × D+b × D^2^, where a and b are constants and D is the dose). The single-step creation of DSBs (a × D) appears to be dominant at lower radiation doses (Figure 3B), while the two-step creation of DSBs (b × D^2^) is more influential as the dose increases (Figure 2B), whereupon the single-step process becomes negligible. Our simple simulation (Figure S3C), which includes rare single-step DSBs (a × D) and frequent two-step DSBs (b × D^2^), has a profile similar to that shown in Figure 2B. In addition, the linear inhibitory effect at low irradiation doses (Figure 3B) demonstrates that the radiation dose can directly generate DSBs. This is also the case for heavy ion irradiation (Figure 5D and E).

It is also interesting to consider the *in vivo* situation. To detect DSBs in eukaryotic cells, immunostaining to detect serine-139 phosphorylation in histone H2Ax (γH2AX) is commonly used [29] [30], although the percentage of DSBs detected as γH2AX foci and their direct relationship remain unclear [52]. A number of studies have demonstrated that γH2AX foci occur frequently in euchromatin regions, and less frequently in heterochromatic regions [51] [53] [54] [55] [56]. Furthermore, it was demonstrated that hypotonic treatment of cells, in which the chromatin is presumably decondensed, produced more γH2AX foci [51]. Although these *in vivo* results reflect many indirect effects, making it difficult to draw simple conclusions, they appear to be in good agreement with our *in vitro* findings. For further *in vivo* study, the use of an ion microbeam [57] or UV laser microirradiation [58] would be useful, since both allow the targeted irradiation of heterochromatic and euchromatic nuclear areas and may enable a direct comparison of the generation of DSBs in these compartments either by a TUNEL assay or the identification of γH2AX.

**Figure 6:**
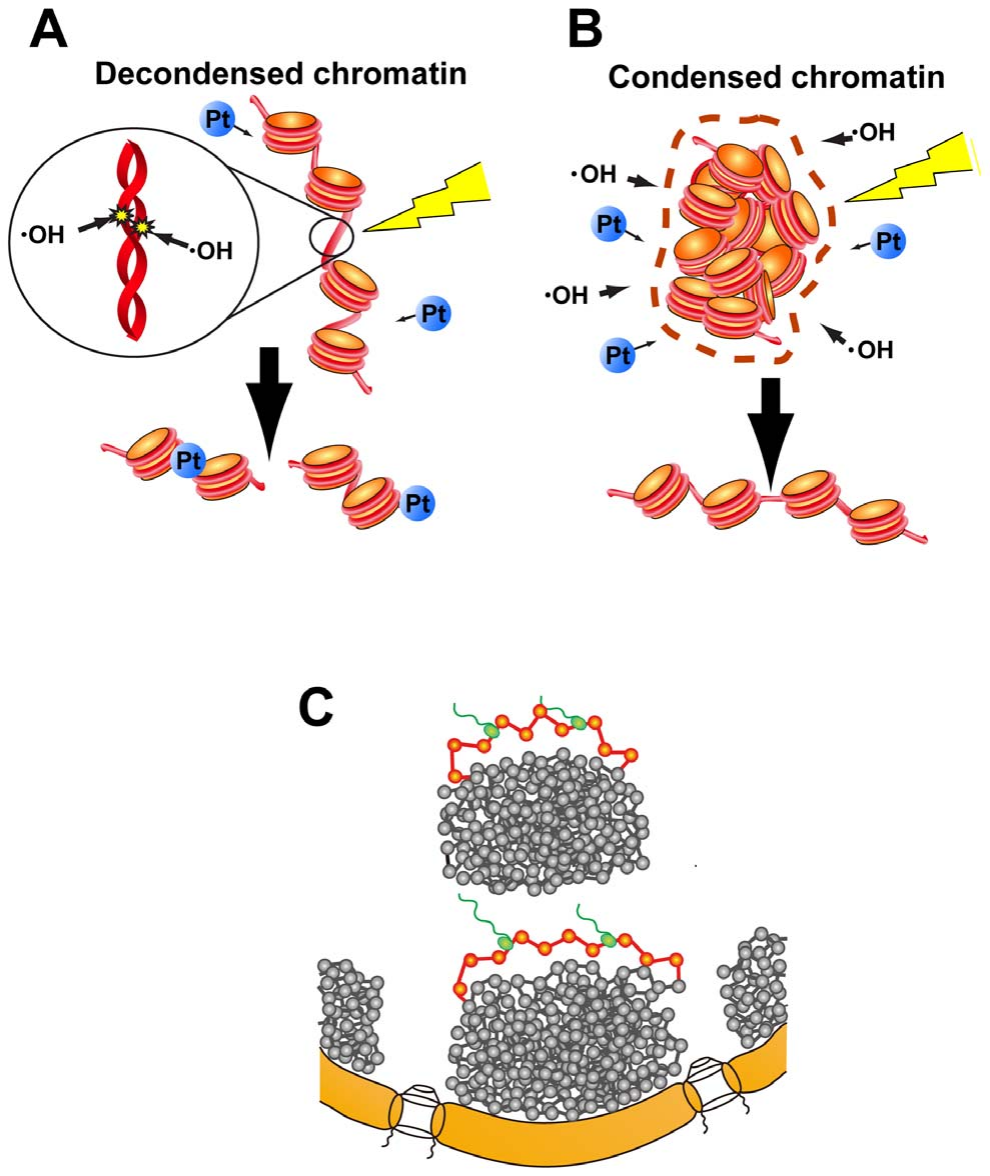
Damage suppression and the compact chromatin domain model. (**A**) Reactive radicals arising from radiolysis of water molecules by irradiation are major contributors to the damaging of decondensed chromatin. In addition, decondensed chromatin is more accessible to chemicals (marked with “Pt”). (**B**) A higher density of chromatin with fewer water molecules means that there is less risk of being attacked by hydroxyl radicals. The situation is also effective at protecting DNA from the binding of cisplatin. (**C**) The proposed compact chromatin domain model. We assume that the condensed state is the “default” and that only the transcribed region, which is decondensed and is sensitive to radiation damage, might loop out from the domain or from the surface of the domain, so as to minimize the risk of radiation or chemical damage.

Since we demonstrated that condensed chromatin has fewer DSBs induced by γ-rays and heavy ions, and is less susceptible to attack by chemical agents such as cisplatin, the present study indicates that chromatin compaction is advantageous, even in interphase cells (Figure 6A and B). This is consistent with the previously proposed idea of a chromatin domain model [5] [9] [13] [14] [18] [59] (Figure 6C). In this model, interphase chromatin forms compact chromatin domains. This compact state is the “default”. The only transcribed region, which is minimally decondensed and could be sensitive to radiation or other types of damage, might be looped out from the domain or at the surface of the domain (Figure 6C). This type of genomic organization could be beneficial for cells, especially in the long run.

We propose that cellular protection by genomic DNA compaction represents a universally conserved function, from viruses to animals, aimed at maintaining genome integrity, because DNA condensation by polyamine was also observed to suppress DSB induction by γ-ray irradiation in previous *in vitro* studies, including our own [25] [26] [27] [28]. The maintenance of genomic DNA integrity by greater compaction would carry a selective advantage during the evolution of prokaryotes. This selective advantage might have even been more important in early evolutionary times when DNA repair mechanisms were preliminary. In eukaryotic organisms, the maintenance of genetic information would be especially critical in germline cells for the next generation. For example, yeast spores have ∼10-fold more condensed genomic DNA than somatic cells [60]. Human primary oocytes, which take 20–40 years to complete meiosis I [61], have compact chromosomes, while mouse and rat have highly extended (dictyate-type) chromatin in their primary oocytes [62]. Surprisingly, human primary oocytes are much more resistant to ionizing radiation than mouse or rat primary oocytes [62], which supports our hypothesis.

Although we emphasized the importance of chromatin compaction in the maintenance of genomic DNA integrity, chromatin compaction might interfere with DNA repair due to a reduction in chromatin accessibility. This might be partly true [39]; however, we recently observed local nucleosome fluctuations in living mammalian cells and demonstrated that this fluctuation increases chromatin accessibility, especially in compact chromatin regions [63] [64]. This novel mechanism to facilitate chromatin accessibility would play an important role in the DNA repair process in compact chromatin domains. Furthermore, it was reported that chromatin decondenses after DSB induction, increasing chromatin accessibility [58].

Finally, it is important to emphasize that our findings provide a theoretical basis for various novel combinations of cancer therapies [65] [66] [67] [68]. Since decondensed chromatin has greater susceptibility to γ-rays, heavy ions, and chemicals, histone deacetyltransferase inhibitors and similar drugs (which decondense chromatin) would greatly enhance the cytotoxic effects of cisplatin and other DNA-damaging drugs in cancer cells, as well as those of radiation therapy involving γ-rays or heavy ions.

## Methods

### Isolation of Nuclei and Chromosomes

For the isolation of nuclei, HeLa cells were maintained in Dulbecco’s modified Eagle’s medium (Invitrogen, Carlsbad, CA, USA) containing 5% fetal bovine serum albumin (BSA; Nichirei Biosciences Inc., Tokyo, Japan) at 37°C in a 5% CO_2_ atmosphere. Collected cells were suspended in nuclei isolation buffer (3.75 mM Tris-HCl [pH 7.5], 20 mM KCl, 0.5 mM EDTA, 0.05 mM spermine, 0.125 mM spermidine, 0.1% Trasylol, 0.1 mM phenylmethylsulphonyl fluoride [PMSF]) and centrifuged at 193 ×*g* for 7 min at room temperature. The cell pellets were resuspended in nuclei isolation buffer and again centrifuged at 193 ×*g* for 7 min at room temperature. The cell pellets were then resuspended in nuclei isolation buffer containing 0.05% Empigen (nuclei isolation buffer+) and homogenized immediately with ten downward strokes using a tight Dounce-pestle. The cell lysates were centrifuged at 433 ×*g* for 5 min. The nuclei pellets were washed in nuclei isolation buffer+ and stored at –20°C in nuclei isolation buffer+ containing 50% glycerol. Chromosome isolation was performed as described previously [45].

### Solid-phase Chromatin Manipulation

Isolated nuclei or chromosomes were suspended in HM buffer (10 mM HEPES-KOH [pH 7.4] and 5 mM MgCl_2_) and attached to poly-l-lysine-coated coverslips by centrifugation at 400 ×*g* for 5 min. For decondensed chromatin, the nuclei or chromosomes on the coverslips were gently transferred to the buffer that contained 1 mM EDTA (pH 8.0). For recondensation, the decondensed chromatin on the coverslip was placed in HM buffer.

### Irradiation with γ-rays of Cobalt-60 (high dose) and Cesium-137 (Low Dose)

For high-dose irradiation (all experiments, with the exception of that shown in Figure 3), condensed, decondensed, or recondensed chromatin was irradiated with cobalt-60 γ-rays at 40 Gy/min at the Radiation Research Center, Osaka Prefecture University (Osaka, Japan). For low-dose irradiation (Figure 3), we irradiated the same sets of samples with cesium-137 at 1.67 Gy/min at the irradiation facility of the National Institute of Genetics (NIG; Mishima, Japan). In both irradiation experiments, the coverslips with chromatin were put in a 12-well cell culture plate (Corning Inc. Life Sciences, Tewksbury, MA, USA) with the indicated buffers and irradiated at room temperature. The applied dose was determined using a Fricke dosimeter.

### TUNEL Assay

After γ-ray irradiation, the condensed, decondensed, or recondensed chromatin on the coverslips was placed in HM buffer, followed by HMK buffer (10 mM HEPES-KOH [pH 7.4], 1 mM MgCl_2_, and 100 mM KCl), to ensure that the chromatin condensation state was uniform. The chromatin samples were fixed with 1% formaldehyde in HMK buffer at room temperature for 15 min. After washing with 50 mM glycine in HMK buffer and then HMK buffer, the samples were stored in HEN buffer (10 mM HEPES-KOH [pH 7.5], 1 mM EDTA, and 100 mM NaCl) at 4°C until use.

DNA damage was detected using a Click-iT TUNEL Alexa Fluor Imaging Assay Kit (Invitrogen) according to the manufacturer’s instructions. The fluorescently labeled samples were co-stained with DAPI to visualize DNA then mounted in PPDI (10 mM HEPES-KOH [pH 7.5], 1 mM MgCl_2_, 100 mM KCl, 80% glycerol, and 1 mg/ml paraphenylene diamine). For the high-dose irradiation samples (Figures 2, 4 and 5), microscopic images to quantify the TUNEL assay signals were acquired under the same imaging conditions using an ECLIPSE E800 fluorescence microscope with a 60× objective lens (Nikon, Tokyo, Japan). For the lower dose irradiation samples (Figure. 3), we used Nikon microscope system Ti-E with a 60× objective lens (Nikon, Tokyo, Japan) using Evolve512 EM-CCD camera (Roper Scientific, USA). The TUNEL assay signals (Alexa 488) were analyzed using NIS-elements BR 3.10 software (Nikon) as follows. The nuclear or chromosomal regions were extracted based on a threshold value of DAPI signal intensity. The mean intensity values of Alexa 488 signals in the extracted nuclear or chromosomal regions were then examined. The mean intensity value in the regions at 0 Gy was used as background. After subtraction of the background signal, the obtained signal intensity values were normalized against that of decondensed chromatin irradiated at 500 Gy. The comet assay was performed according to the manufacturer’s instructions (Trevigen, Gaithersburg, MD, USA).

### Nuclear and Chromosomal Volume Measurements

After fixation with 1% formaldehyde, the samples were washed with 50 mM glycine and stained with 2 nM TO-PRO-3 solution (Invitrogen) at 37°C for 30 min. After washing, Z-stack images were acquired using a LSM510 META laser scanning confocal microscope (Carl Zeiss, Wetzlar, Germany) with a 100× objective at 0.48-µm intervals. The obtained images were processed using the LSM Image Browser (Carl Zeiss) and ImageJ software (NIH, Bethesda, MD, USA).

### Protein Composition and Concentration Analyses

Condensed, decondensed, and recondensed nuclei or chromosomes were collected by centrifugation (3000 rpm, 10 min). The pellets, which contained ∼1.26 mg of DNA, were completely dissolved in Laemmli sodium dodecyl sulfate (SDS) sample buffer by sonication. After boiling at 95°C for 5 min, the lysates were subjected to 10–20% gradient SDS-polyacrylamide gel electrophoresis (PAGE). To quantify the protein levels, BSA was used as a standard. The gel was stained with Coomassie brilliant blue R-250 (CBB) and the image was acquired using an LAS-1000 imaging system (Fujifilm, Tokyo, Japan). A quantitative analysis was performed using ImageJ software.

### Simulation

The simulation was conducted as follows using Microsoft Visual C++. Two random numbers (SSBs) from 1 to 100,000 (100 kb) were generated independently for strands a and b (Figure S3A). If the difference between the numbers on the two strands was less than 10, we counted the pair as a DSB (Figure S3A). We repeated this process at the indicated times and plotted the number of DSBs against the number of SSBs (Figure S3B). The number of created DSBs was quadratically increased with the number of generated SSBs. For Figure S3C, two random numbers (SSBs) from 1 to 100000 (100 kb) were again generated independently for strands a and b. Every 50 SSBs, a DSB was also created at random.

### Carbon Ion Beam Irradiation

Heavy ion treatment was performed using the Heavy Ion Medical Accelerator in Chiba (HIMAC) at the National Institute of Radiological Sciences (Chiba, Japan). The accelerated ions used in this study were carbon ions (290 MeV/n). Details concerning the characteristics of the carbon ion beams, biological irradiation procedures, and dosimetry may be found elsewhere [69] [70].

### Cisplatin Treatment of Condensed and Decondensed Chromatin

Isolated nuclei (∼1×10^7^) were suspended in HM buffer or 1 mM EDTA containing buffer and treated with cisplatin at 2 mM overnight at room temperature. After five washes with HMK buffer, half of the samples were subjected to ICP-MS (chromatin fractions). DNA was extracted from the remaining samples and subjected to ICP-MS (DNA fractions). We performed ICP-MS using ELAN DRC II (PerkinElmer, Waltham, MA, USA).

## Supporting Information

Figure S1 **Schematic representation of the terminal deoxynucleotidyl transferase (TdT) dUTP nick end labeling (TUNEL) assay.** **(A)** EdUTPs are directly incorporated into DSB sites in the chromatin by TdT. With the Click reaction, fluorescent azides transfer to the EdUTPs, thereby labeling the DSBs. **(B)** Upon mild DNase I treatment, the nuclei acquire fluorescent DSB signals, as assessed in the TUNEL assay.
(TIF)

Figure S2 **DSB detection using the comet assay.** The relative tail lengths of the comets in the condensed **(**C**)** and decondensed **(**D**)** nuclei are shown as bar graphs. The error bar shows the standard deviation. Decondensed chromatin **(**D**)** is more sensitive to γ-ray irradiation than compact chromatin **(**C**)**.
(TIF)

Figure S3 **Simulation of DSB induction.** **(A)** Simulation scheme: DSBs occur when two random SSBs are generated within ten bases of each other in the double strands of the DNA. **(B)** The numbers of created DSBs and SSBs are plotted. The number of created DSBs increased quadratically with the number of generated SSBs. **(C)** Similar to **A** and **B**, two random numbers (SSBs) from 1 to 100000 (100 kb) were generated independently for strands a and b. For every 50 SSBs, a DSB was also created at random. The numbers of created DSBs and SSBs are plotted.
(TIF)

Figure S4 **Protein compositions of condensed, decondensed, and recondensed chromatin.** **(A)** Condensed, decondensed, and recondensed nuclei were electrophoresed on gradient SDS-PAGE gels and stained with CBB. **(B)** The total, histone, and non-histone fractions were quantified and are shown as bar graphs. N = 3. Error bars show the standard deviation.
(TIF)

Figure S5 **Protein compositions of the decondensed nuclei before and after irradiation.** **(A)** Protein samples of decondensed nuclei before and after irradiation were electrophoresed on gradient SDS-PAGE gels and stained with CBB. **(B)** Total, histone, and non-histone fractions were quantified and shown as bar graphs. Error bars show the standard deviation.
(TIF)

Figure S6 **Protein composition of condensed, decondensed, and recondensed chromosomes.** **(A)** Samples of condensed, decondensed, and recondensed chromosomes were electrophoresed on gradient SDS-PAGE gels and stained with CBB. **(B)** The total, histone, and non-histone fractions were quantified and are shown as bar graphs. Error bars show the standard deviation.
(TIF)

Figure S7 **Nuclear volumes of condensed and decondensed nuclei in the presence of PEG or DTT.** **(A)** Microscopic images of condensed and decondensed nuclei in the presence of PEG or DTT (DNA staining). Bar, 10 µm. **(B)** The nuclear volumes of the condensed and decondensed nuclei in the presence of PEG or DTT are shown as bar graphs. Error bars show the standard deviation.
(TIF)
