# Supplementary figures and images for: Chromatin Compaction Protects Genomic DNA from Radiation Damage
Source: PLoS One. 2013 Oct 9;8(10):e75622. doi: 10.1371/journal.pone.0075622 (PMC3794047; doi:10.1371/journal.pone.0075622)

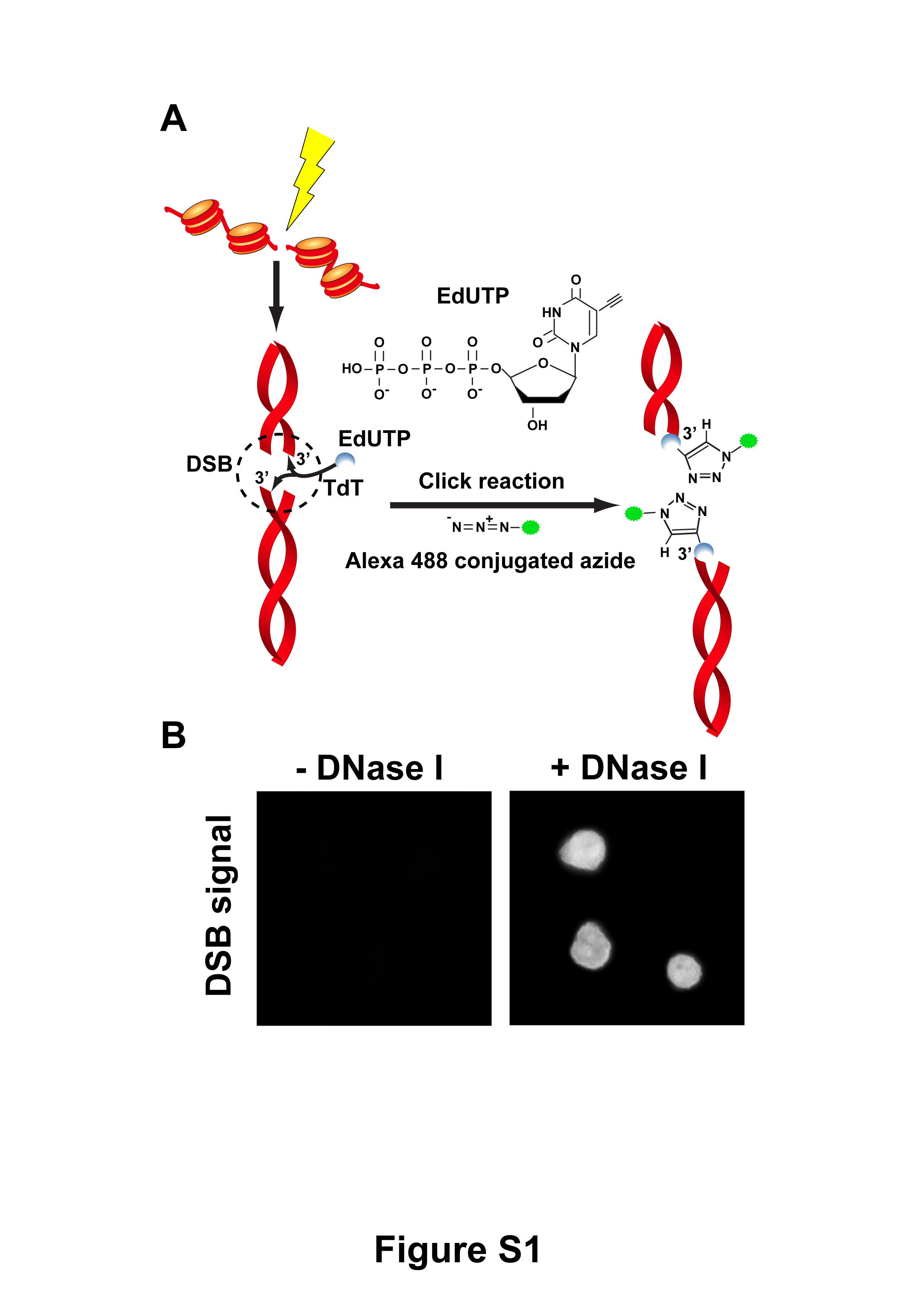

Supplement: Figure S1 — Schematic representation of the terminal deoxynucleotidyl transferase (TdT) dUTP nick end labeling (TUNEL) assay. (A) EdUTPs are directly incorporated into DSB sites in the chromatin by TdT. With the Click reaction, fluorescent azides transfer to the EdUTPs, thereby labeling the DSBs. (B) Upon mild DNase I treatment, the nuclei acquire fluorescent DSB signals, as assessed in the TUNEL assay. (TIF) [file pone.0075622.s001.tif]

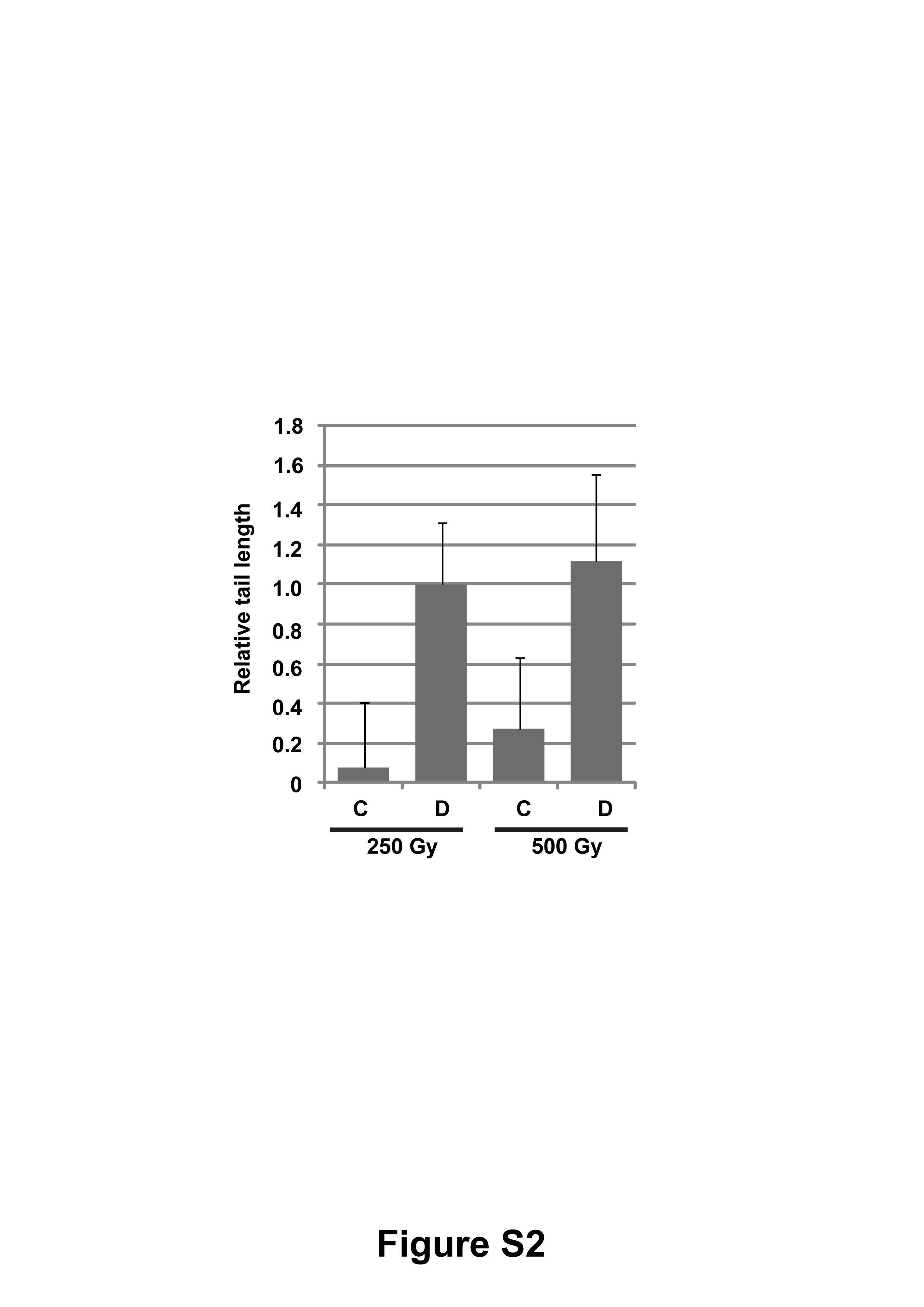

Supplement: Figure S2 — DSB detection using the comet assay. The relative tail lengths of the comets in the condensed (C) and decondensed (D) nuclei are shown as bar graphs. The error bar shows the standard deviation. Decondensed chromatin (D) is more sensitive to γ-ray irradiation than compact chromatin (C). (TIF) [file pone.0075622.s002.tif]

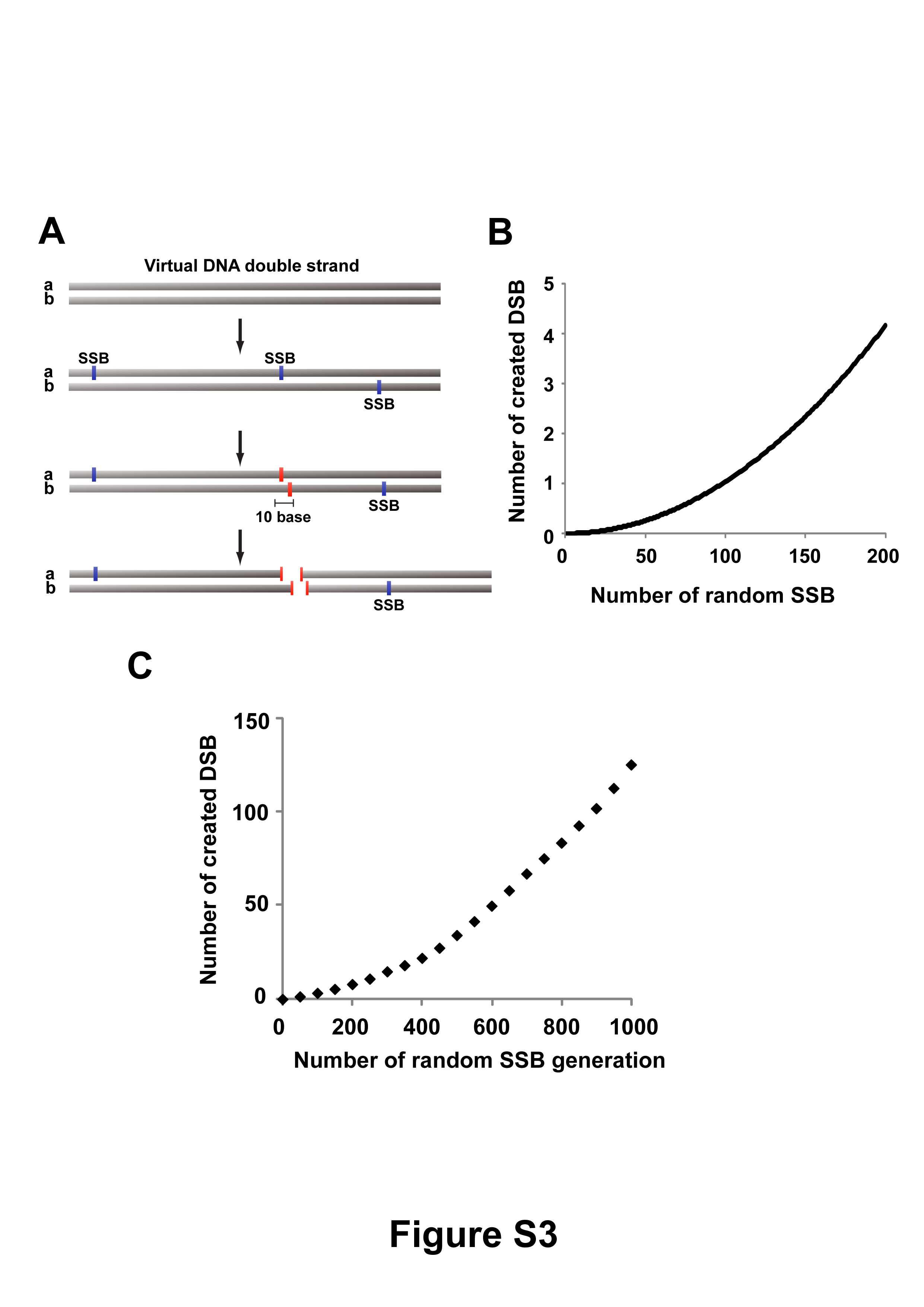

Supplement: Figure S3 — Simulation of DSB induction. (A) Simulation scheme: DSBs occur when two random SSBs are generated within ten bases of each other in the double strands of the DNA. (B) The numbers of created DSBs and SSBs are plotted. The number of created DSBs increased quadratically with the number of generated SSBs. (C) Similar to A and B, two random numbers (SSBs) from 1 to 100000 (100 kb) were generated independently for strands a and b. For every 50 SSBs, a DSB was also created at random. The numbers of created DSBs and SSBs are plotted. (TIF) [file pone.0075622.s003.tif]

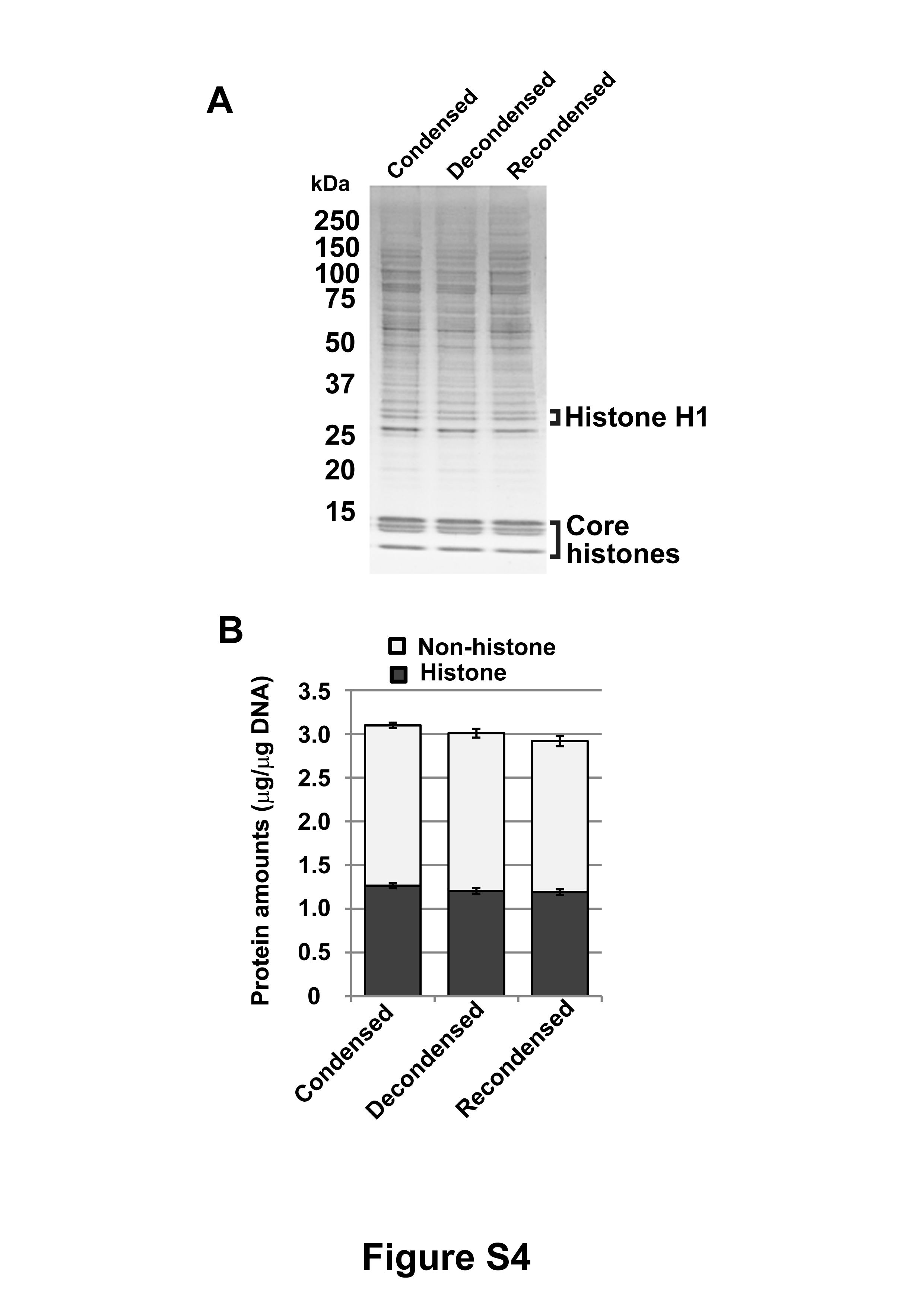

Supplement: Figure S4 — Protein compositions of condensed, decondensed, and recondensed chromatin. (A) Condensed, decondensed, and recondensed nuclei were electrophoresed on gradient SDS-PAGE gels and stained with CBB. (B) The total, histone, and non-histone fractions were quantified and are shown as bar graphs. N = 3. Error bars show the standard deviation. (TIF) [file pone.0075622.s004.tif]

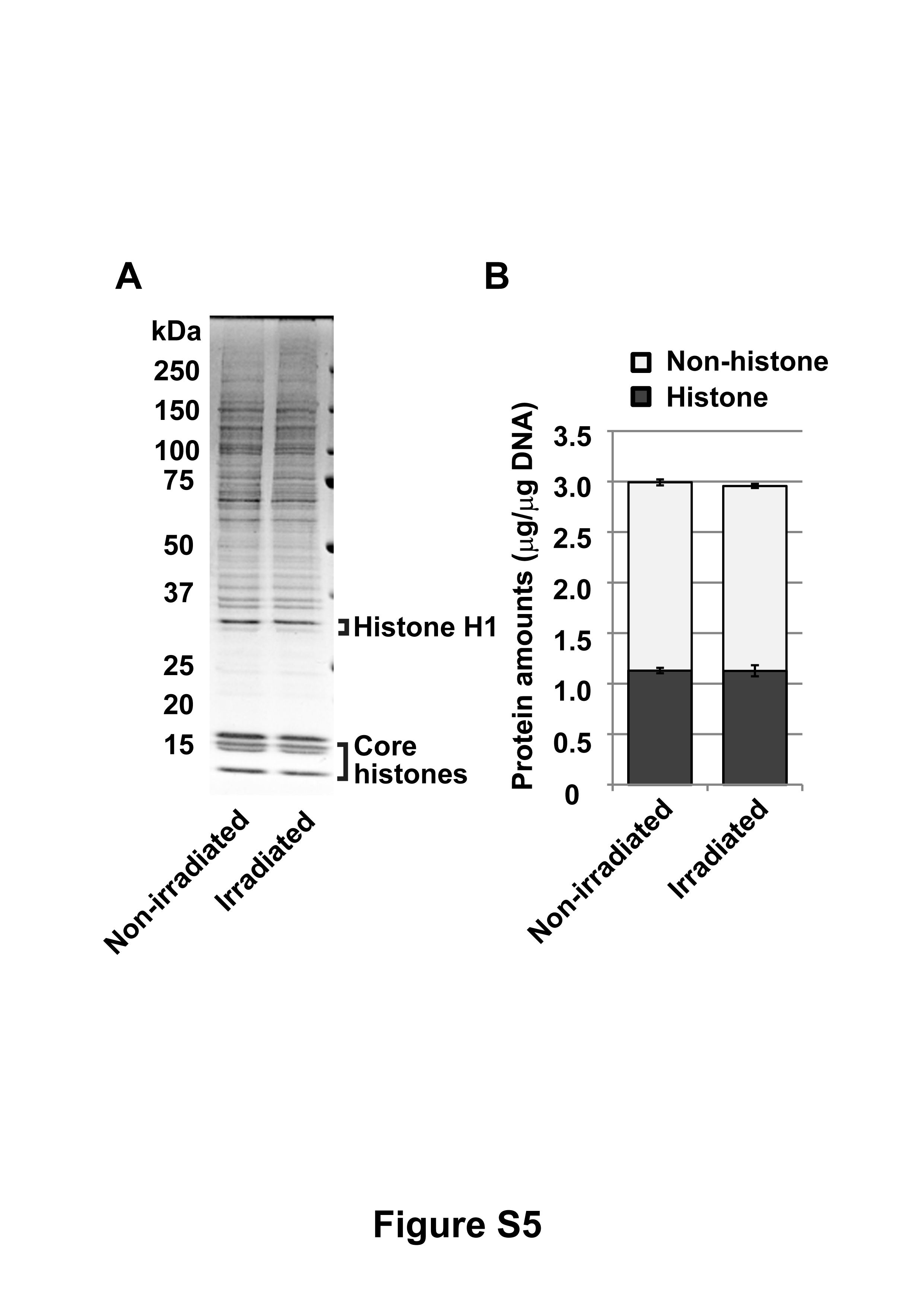

Supplement: Figure S5 — Protein compositions of the decondensed nuclei before and after irradiation. (A) Protein samples of decondensed nuclei before and after irradiation were electrophoresed on gradient SDS-PAGE gels and stained with CBB. (B) Total, histone, and non-histone fractions were quantified and shown as bar graphs. Error bars show the standard deviation. (TIF) [file pone.0075622.s005.tif]

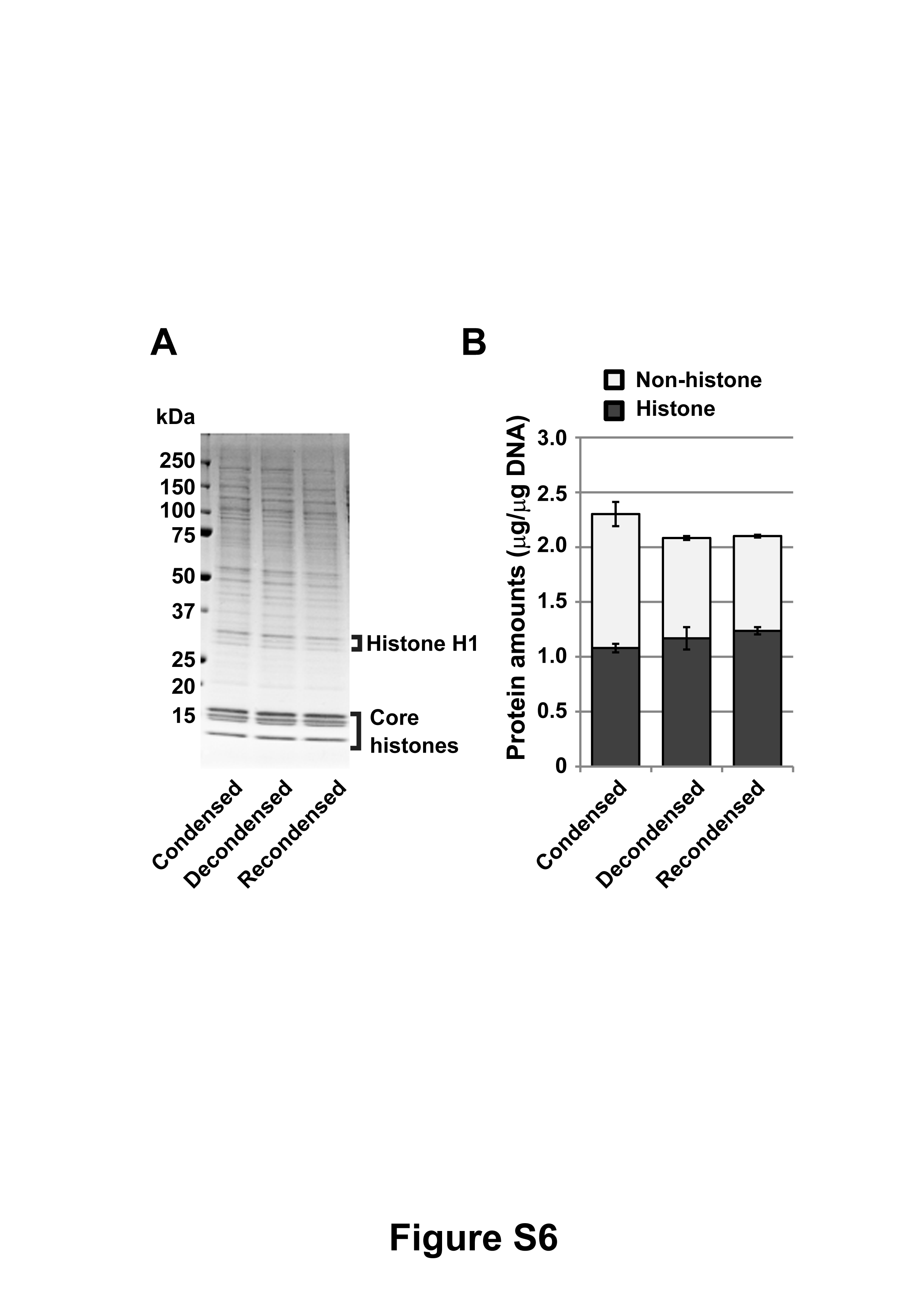

Supplement: Figure S6 — Protein composition of condensed, decondensed, and recondensed chromosomes. (A) Samples of condensed, decondensed, and recondensed chromosomes were electrophoresed on gradient SDS-PAGE gels and stained with CBB. (B) The total, histone, and non-histone fractions were quantified and are shown as bar graphs. Error bars show the standard deviation. (TIF) [file pone.0075622.s006.tif]

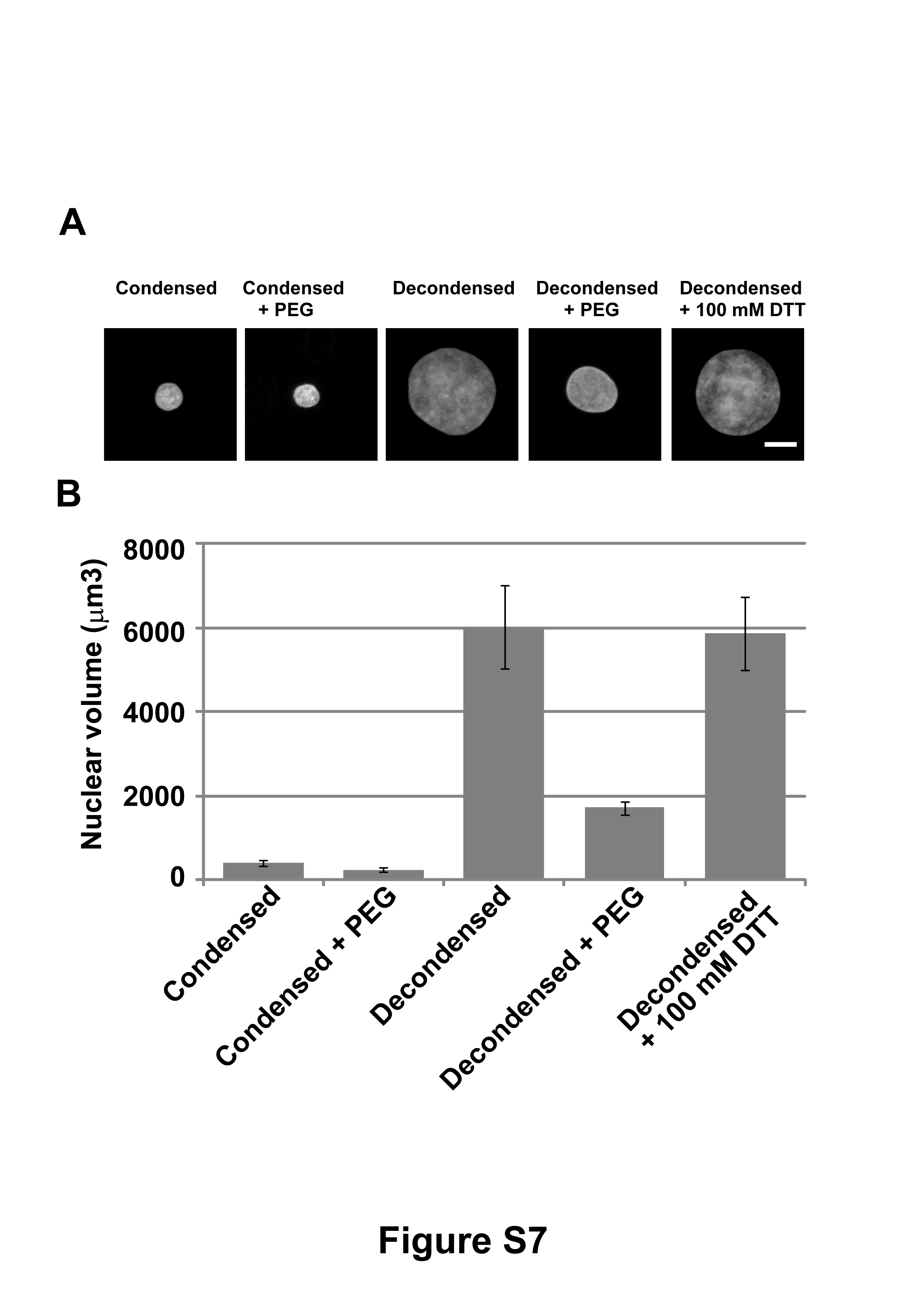

Supplement: Figure S7 — Nuclear volumes of condensed and decondensed nuclei in the presence of PEG or DTT. (A) Microscopic images of condensed and decondensed nuclei in the presence of PEG or DTT (DNA staining). Bar, 10 µm. (B) The nuclear volumes of the condensed and decondensed nuclei in the presence of PEG or DTT are shown as bar graphs. Error bars show the standard deviation. (TIF) [file pone.0075622.s007.tif]
